# Phosphodiesterase 4D activity in acrodysostosis-associated neural pathology: too much or too little?

**DOI:** 10.1093/braincomms/fcae225

**Published:** 2024-06-29

**Authors:** Oliver F W Gardner, Tianshu Bai, George S Baillie, Patrizia Ferretti

**Affiliations:** Developmental Biology and Cancer Department, University College London Great Ormond Street Institute of Child Health, London WC1N 1EH, UK; Developmental Biology and Cancer Department, University College London Great Ormond Street Institute of Child Health, London WC1N 1EH, UK; School of Cardiovascular & Metabolic Health, University of Glasgow, Glasgow G12 8QQ, UK; Developmental Biology and Cancer Department, University College London Great Ormond Street Institute of Child Health, London WC1N 1EH, UK

**Keywords:** acrodysostosis, PDE4D, cAMP, protein kinase A

## Abstract

Members of the phosphodiesterase 4 (PDE4) enzyme family regulate the availability of the secondary messenger cyclic adenosine monophosphate (cAMP) and, by doing so, control cellular processes in health and disease. In particular, PDE4D has been associated with Alzheimer’s disease and the intellectual disability seen in fragile X syndrome. Furthermore, single point mutations in critical PDE4D regions cause acrodysostosis type 2(ACRDYS2, also referred to as inactivating PTH/PTHrP signalling disorder 5 or iPPSD5), where intellectual disability is seen in ∼90% of patients alongside the skeletal dysmorphologies that are characteristic of acrodysostosis type 1 (ACRDYS1/iPPSD4) and ACRDYS2. Two contrasting mechanisms have been proposed to explain how mutations in PDE4D cause iPPSD5. The first mechanism, the ‘over-activation hypothesis’, suggests that cAMP/PKA (cyclic adenosine monophosphate/protein kinase A) signalling is reduced by the overactivity of mutant PDE4D, whilst the second, the ‘over-compensation hypothesis’ suggests that mutations reduce PDE4D activity. That reduction in activity is proposed to cause an increase in cellular cAMP, triggering the overexpression of other PDE isoforms. The resulting over-compensation then reduces cellular cAMP and the levels of cAMP/PKA signalling. However, neither of these proposed mechanisms accounts for the fine control of PDE activation and localization, which are likely to play a role in the development of iPPSD5. This review will draw together our understanding of the role of PDE4D in iPPSD5 and present a novel perspective on possible mechanisms of disease.

## Teaser

Gardner *et al*. provide an innovative perspective on the role of phosphodiesterase 4D (PDE4D) in the aetiology of acrodysostosis, a rare genetic disease that affects neural and skeletal development. This review discusses the hypotheses that have been presented in the literature and suggests novel disease mechanisms.

## Graphical Abstract

**Graphical Abstract:**
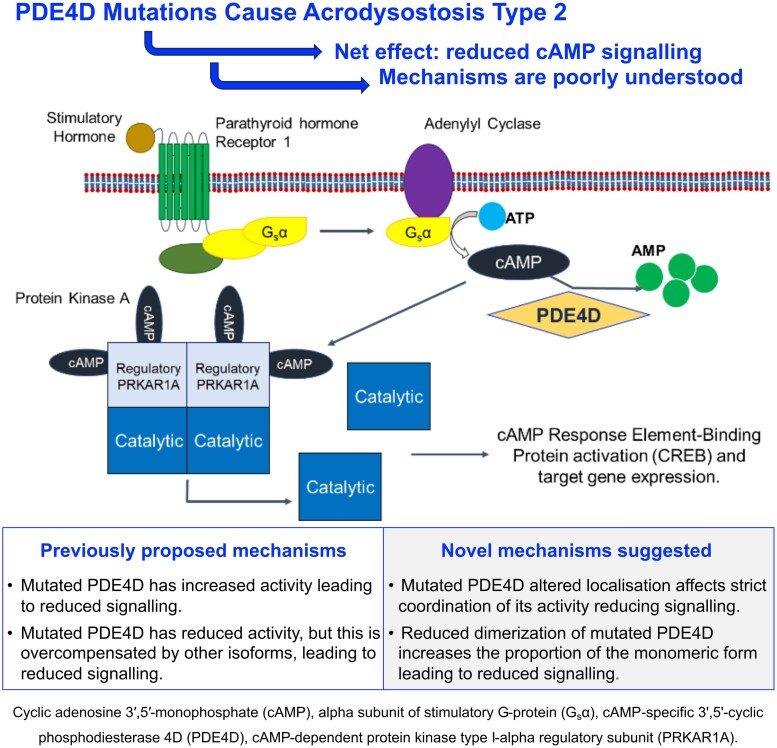

## Phosphodiesterases and the cAMP/PKA signalling pathway

Phosphodiesterases (PDEs) are the only enzymes known to degrade the cyclic nucleotide secondary messenger cAMP (cyclic adenosine monophosphate) and cGMP (cyclic guanosine monophosphate). They are ubiquitously expressed and play a role in many cellular mechanisms and diseases.^1-3^ The mammalian PDE super-family consists of 11 individual families (PDE1–PDE11) that are categorized by their structure, size, enzymatic properties, regulation and specificity for either cAMP, cGMP or both.^4,5^

**Figure 1:**
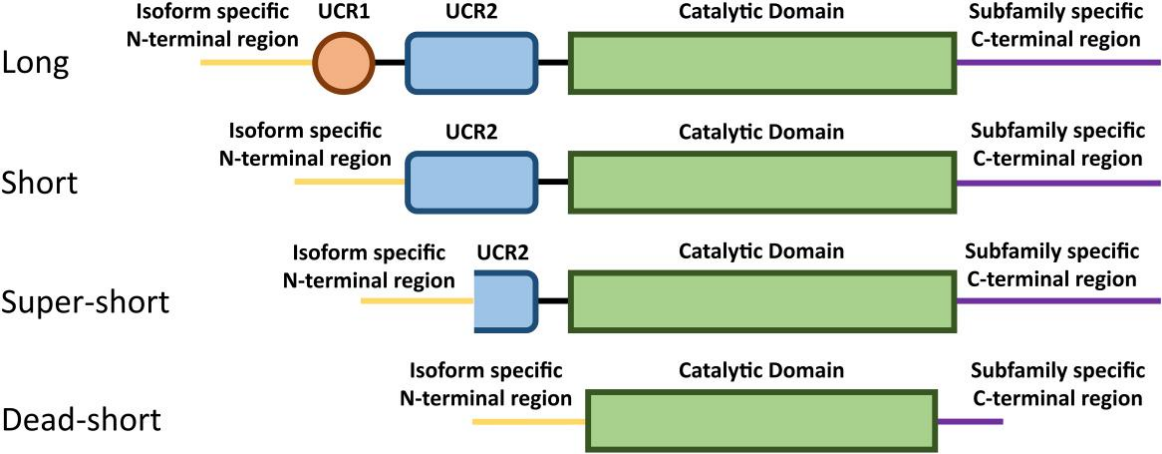
**PDE4 isoforms are categorized by length based on the functional domains that are present in the isoform.** All isoforms contain a highly conserved catalytic domain (truncated in dead-short isoforms), flanked by a combination of structural and regulatory upstream conserved regions (UCR1 and 2), isoform specific N-terminal domain and subfamily specific C-terminal region.

The enzymes of the PDE4 family specifically hydrolyze cAMP and are encoded by four genes, *PDE4A*, *B*, *C* and *D*,^6^ which through different transcriptional start sites and the alternative splicing of mRNA are transcribed into more than 20 isoforms.^4^ Each isoform of the PDE4 family consists of a highly conserved catalytic domain, which is primarily responsible for the hydrolysis of cAMP to AMP but also contains regulatory sites^7^ and interacts with scaffolding proteins important in PDE localization.^8^ All PDE4 isoforms carry a subfamily (A–D) specific C-terminal region and an isoform specific combination of: N-terminal region, upstream conserved regions (UCR1 and/or UCR2) and C-terminal region^6^ (Fig. 1). Each PDE4 isoform has a unique N-terminal region that enables it to localize to different intracellular compartments by binding with different scaffolding proteins.^1,2^ The UCRs are structural and regulatory regions that are unique to PDE4s^9^ and are responsible for the formation of dimers by long isoforms.^10^ In the context of acrodysostosis, disease-causing mutations have been localized across UCR1, UCR2 and the catalytic domain.^11-13^

The isoforms of the PDE4 family are subdivided by length, depending on the number of these functional regions that are included (Fig. 1). Isoforms can be classified as long, short, super-short and dead-short.^5^ All isoforms (except PDE4D2) have an isoform specific N-terminal region, long isoforms then have UCR1, UCR2, catalytic domain and subfamily specific C-terminal. Compared to the long isoforms short isoforms lack UCR1, super-short isoforms lack UCR1 and part of UCR2 and dead-short isoforms have truncated N- and C-terminal regions, no UCR1 or 2 and inactive catalytic domains.^5^

The hydrolytic activity of PDE4s is regulated in a number of ways, including dimerization^14^ and post-translational modification, particularly phosphorylation by protein kinase A (PKA) in UCR1 that leads to a 2–6-fold increase in enzyme activity.^15,16^ PDE4s are also regulated by SUMOylation at the N-terminal end of the catalytic site, which locks long PDE4s in an active state and phosphorylation by extracellular signal-regulated kinase (ERK) at the C-terminal end of the catalytic site, which keeps the dimer in the inactive state.^17^ PDEs can also be activated in response to lipid biding, e.g. the activation in PDE4D3 that results from the binding of phosphatidic acid to the N-terminal domain.^18^

The role of PDEs is to spatially and temporally control the distribution of cAMP/cGMP within cells. To do this, PDEs hydrolyze cAMP/cGMP and therefore prevent them from activating signalling pathways via the four known cAMP effectors, PKA, exchange protein directly activated by cAMP (EPAC), cyclic nucleotide-gated ion channel (CNGC) and Popeye domain containing proteins (POPDC).^2,19-21^ In doing so, PDEs generate strictly controlled intracellular gradients of cyclic nucleotides, which in turn allow these ubiquitous secondary messengers to work simultaneously via different pathways and with different levels of stimulation.^2,22^ The way that this level of control is achieved is via a strict regulation of the sub-cellular localization of PDEs, much of which occurs through the N-terminal targeting domains (which are specific for each isoform) and the docking domain within the catalytic unit.^6,23^ The ability to target specific isoforms in this way facilitates the formation of signalosomes, large protein complexes formed through the interaction of PDEs with cAMP effector proteins, membranes and scaffolding molecules (e.g. AKAP), that allow the cAMP concentration around specific effectors to be highly controlled.^1,24^ This finely tuned control of PDE localization results in the 3D compartmentalization of cAMP in cells^25,26^ and allows for its downstream effects to be tightly regulated so that each receptor activation event that generates cAMP results in an appropriate physiological response.

In this review, we will discuss the role of PDE4D in acrodysostosis and the contrasting mechanisms that have been proposed to explain how mutations in PDE4D reduce cAMP signalling and lead to acrodysostosis.^27^ As PDE4 has been directly implicated in other neurological diseases, the lessons learned here will also be relevant in other contexts.

## PDE4D mutations as a cause of acrodysostosis

Acrodysostosis is as an extremely rare skeletal dysplasia characterized by facial dysostosis and brachydactyly^28,29^ that are also associated with learning difficulties and hormone resistance. Due to its rarity, and potential underdiagnosis, accurate evaluations of the occurrence of acrodysostosis are difficult to make. However, it has been estimated to affect fewer than 1/300 000 White French children^30^ and the authors are only aware of 150 cases with confirmed genetic diagnoses reported in the literature.

There are two separate forms of the disease, each caused by mutations in either the *PRKAR1A* (cAMP-dependent protein kinase type I-alpha regulatory subunit) or *PDE4D* (cAMP-specific 3′,5′-cyclic phosphodiesterase 4D) genes, both of which are components of the cAMP/PKA signalling pathway. Different names have been used in the literature for the two forms including acrodysostosis with/without hormone resistance and acrodysostosis types 1 and 2. In 2016, Thiele *et al*.^27^ classified acrodysostosis as an inactivating parathyroid hormone/parathyroid hormone-related peptide signalling disorder (iPPSD), based on shared characteristics with other diseases that affect the PTH/PTHrP and cAMP/PKA signalling pathways e.g. pseudohypoparathyroidism (PHP). In this review, we will use the iPPSD nomenclature; specifically, we will use iPPSD4 to refer to acrodysostosis caused by mutations in *PRKAR1A* and iPPSD5 for acrodysostosis caused by mutations in *PDE4D*. We will continue to use acrodysostosis when referring to both forms of the disease at the same time.

Both forms of acrodysostosis affect males and females equally^31^ and present with brachydactyly and a depressed nasal bridge.^11,31^ Most patients with *PDE4D* mutations also have nasomaxillary hypoplasia^11^ and intellectual disability,^11,31,32^ which is rarely observed when *PRKAR1A* is mutated. Patients with mutations in *PRKAR1A* usually present with hormone resistance^11,33-35^ (although this is not unique to those with *PRKAR1A* mutations^35^), short stature^31^ and small size for gestational age at birth.^36^

**Figure 2:**
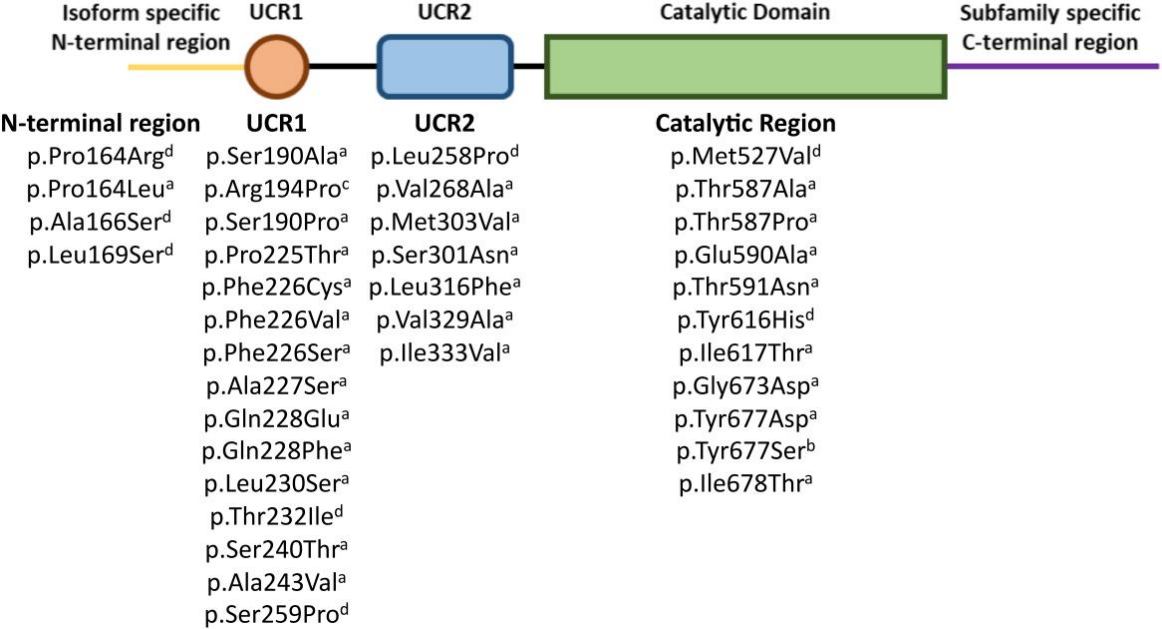
**The location of known iPPSD5 disease-causing mutations in the long form of phosphodiesterase 4D (PDE4D).**  ^a^Denotes mutations first summarized by Michot *et al*.,^11^  ^b^identifies the mutation described by Zhan *et al*.,^83^  ^c^identifies the mutation described by Petraityte *et al*.^84^ and ^d^denotes mutations first described by Ertl *et al*.^36^ UCR1 and 2, upstream conserved regions.

The majority of acrodysostosis patients carry *de novo* mutations,^11^ although familial cases have been described.^11,34,37^ Neither the mutations in *PDE4D* or *PRKAR1A* are found at a specific locus within the genes, instead they are spread throughout the functional domains of the proteins, specifically the catalytic domain and UCR1 and UCR2 in *PDE4D* (Fig. 2) and the cAMP binding domains in *PRKAR1A*.^11,31,32,34,35^ Many of the mutations that have been described in acrodysostosis patients are unique and not shared by other patients; the only recurrent mutation that has been well described is the Arg368*Del mutation in *PRKAR1A*, which leads to a 14 amino acid truncation of the protein and was the first mutation linked to acrodysostosis.^38^

Although there are a wide array of mutations known to cause iPPSD4, they cluster in the two cAMP binding domains of PRKAR1A, providing a common mechanism of action. However, in iPPSD5 known mutations are spread through different functional domains of the gene (Fig. 2), raising questions about how they could have the same ultimate effect on the function of the enzyme. Work by Cedervall *et al*.^10^ has shown that in the final folded form of PDE4D protein iPPSD5 mutations, which are widely spread through the gene sequence, cluster in the hinge region between the autoinhibitory and dimerization domains, or at the interface between the catalytic and autoinhibitory domains. This clustering of mutations, combined with the PDE4 structure that suggests self-regulation of activity, provides a clear rationale for a common mechanism of action between widely spaced mutations.

## PKA signalling and inactivating parathyroid hormone/parathyroid hormone-related peptide signalling disorders

**Figure 3:**
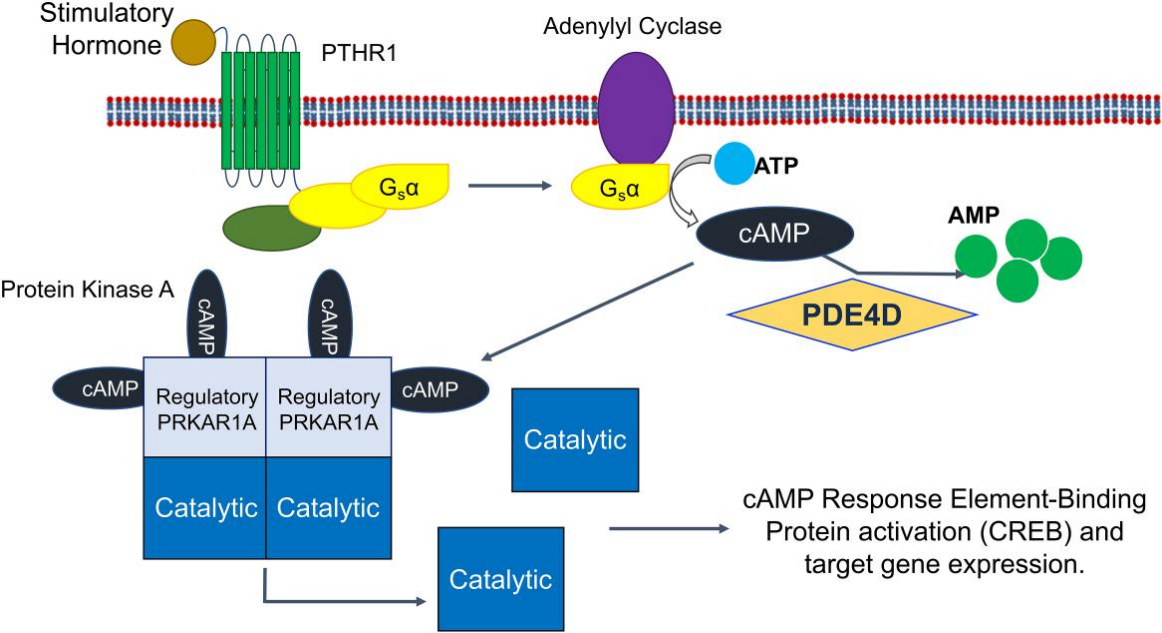
**The cAMP/PKA/CREB signalling pathway.** Agonist binding to the PTHR1 seven transmembrane G-protein coupled receptor leads to activation and release of the alpha subunit of stimulatory G-protein, which activates adenylyl cyclase, leading to the generation of cAMP from ATP. cAMP can then bind to the regulatory subunit of PKA (PRKAR1A), leading to the release and activation of the catalytic subunit that activates the transcription factor CREB by phosphorylation, inducing PKA associated gene expression. PDEs (here PDE4D) remove cAMP from the pathway via hydrolysis to AMP, decreasing signalling through the pathway. AMP, adenosine monophosphate; ATP, adenosine triphosphate; cAMP, cyclic adenosine monophosphate; CREB, cAMP response element-binding protein; G_s_α, alpha subunit of stimulatory G-protein; PDE, phosphodiesterase; PDE4D, cAMP-specific 3′,5′-cyclic phosphodiesterase 4D; PKA, protein kinase A; PRKAR1A, cAMP-dependent protein kinase type I-alpha regulatory subunit; PTHR1, parathyroid hormone receptor 1.

The route by which all mutations causing iPPSD have their effect is by reducing the signalling through the PTH/PTHrP mediated cAMP/PKA pathway.^27^ Signalling in this pathway is initiated by the binding of PTH or PTHrP to the parathyroid hormone 1 receptor (PTH1R) (Fig. 3), a G-protein coupled receptor. This activates and releases the alpha subunit of stimulatory G-protein, which in turn activates adenylyl cyclase.^4,39,40^ Adenylyl cyclase converts ATP to cAMP, which is then free to diffuse through the cell and activate four types of effector protein: PKA, EPAC, CNGC and POPDC.^2,19,20^ In the case of iPPSDs and acrodysostosis, the relevant effector protein is PKA. In its inactive basal state, PKA is a tetramer consisting of two regulatory and two catalytic subunits. Once generated by adenylyl cyclase, a molecule of cAMP first binds to the to the cyclic AMP binding domain-B (CBD-B) of each of the two regulatory subunits, followed by binding of a further cAMP molecules to the CBD-A domains, which triggers the release of the two active catalytic subunits.^41,42^ Once active, the PKA catalytic subunit is free to phosphorylate a range of proteins (including PDEs^10^), but in the PTH/PTHrP pathway primarily acts to phosphorylate Ser133 of the transcription factor CREB (cAMP response element-binding protein), which mediates downstream gene expression through binding to cAMP Responsive Elements associated with target genes.^43,44^

Both iPPSD4 and iPPSD5 result from mutations that lead to a reduction in the amount of signalling through the cAMP/PKA pathway.^45,46^ In iPPSD4, it is well accepted that mutations in the cAMP binding regions of PKAR1A reduce cAMP binding and therefore prevent the release and activation of the catalytic PKA subunit, reducing activation of the transcription factor CREB by phosphorylation and therefore lowering downstream gene induction.^32,38,41,42,47,48^

In contrast, despite investigations by several groups, the mechanism by which mutations in PDE4D leads to a reduction in cAMP/PKA signalling, and therefore iPPSD5, is not well understood.^32,49,50^ As the role of PDEs is to decrease the amount of signalling through the pathway by removing cAMP, it might be expected that iPPSD5 mutations lead to increased PDE4D activity, however evidence has been published that show both increased^50^ and decreased activity of PDE mutants.^32,51^

The key to unlocking this mechanism is to understand the effect that iPPSD causing mutations have on the behaviour of PDE4D, particularly its activity and its localization within cells.

## Contradictory mechanisms have been proposed to explain the role of PDE4D mutations in iPPSD5

There are several strands of indirect evidence that support the assertion that iPPSD5 mutations reduce signalling though the cAMP/PKA pathway. The first is the similarity in clinical presentation between iPPSD5 and iPPSD4 (where the reduction in PKA signalling is understood) as well as other conditions caused by activating mutations in PDEs that have similar presentations, including other iPPSDs^27,45^ and hypertension and brachydactyly syndrome.^52^ Secondly, no iPPSD5 mutations have been described that would significantly disrupt or ablate the expression of PDE4D; it has also been shown that a mutation that leads to haploinsufficiency of PDE4D (5q12.1-haploinsufficiency syndrome) presents with some symptoms that are the opposite of those seen in iPPSD5 e.g. lengthened fingers and nose.^35^ Thirdly, there is significant evidence that PDE4 inhibitors, which cause increased PKA signalling, improve cognitive performance in humans.^53-56^ This contrasts with the intellectual disability seen in patients with iPPSD5, where PKA signalling appears to be reduced. Finally, in one of the only two studies performed on patient derived cells, Kaname *et al*.^32^ showed that Epstein–Barr virus-transformed lymphocytes from iPPSD4 and iPPSD5 patients demonstrated reduced CREB phosphorylation in response to forskolin induced synthesis of cAMP compared to controls, implying a reduced capacity for cAMP/PKA signalling in these cells.^32^

The only work that has suggested that acrodysostosis causing mutations lead to an increase rather than a decrease in PKA signalling was recently published by Venkatakrishnan *et al*.^51^ This article looked at the effect that mutations associated with acrodysostosis in PRKAR1A and PDE8 have on the termination of cAMP induced PKA signalling. Their data showed that these mutations disrupted the removal and hydrolysis of cAMP from PRKAR1A, as a result, delaying the re-association of the regulatory and catalytic subunits, prolonging PKA activation. This suggests that these mutations would lead to increased signalling through the PKA pathway, rather than the decrease in signalling that is currently accepted in the literature. However, it is important to note that his work focused on the effect of iPPSD5 analogous mutations in PDE8, an isoform of PDE that is not known to be associated with iPPSD5 and, despite some similarities, one that has a different structure and regulatory controls to PDE4D. This work also did not consider the effect of these mutations on cAMP/PKA signalling in living cells. Although this work may provide an important insight to the relationship between PRKAR1A and PDEs, more evidence is required to show that this indicates an alternative disease mechanism for iPPSD5.

In many monogenic disorders the identification of a causative mutation in a particular gene reveals a clear mechanism of disease. In iPPSD4, disruption of PTH/PTHrP signalling caused by mutations in *PRKAR1A* provides an explanation for the disease symptoms. The dominant negative effect of mutant PRKAR1A proteins, which directly inhibit the release of the active PKA catalytic subunit, leads to an obvious route for resistance to hormones such as thyroid-stimulating hormone and PTH that act via PTH1R^33,57^ and the skeletal effects of iPSSD5. PTHrP is known to have a crucial role in bone growth, especially the elongation of the long bones^58,59^ that is more affected in iPPSD4 than iPPSD5.^11^

**Figure 4:**
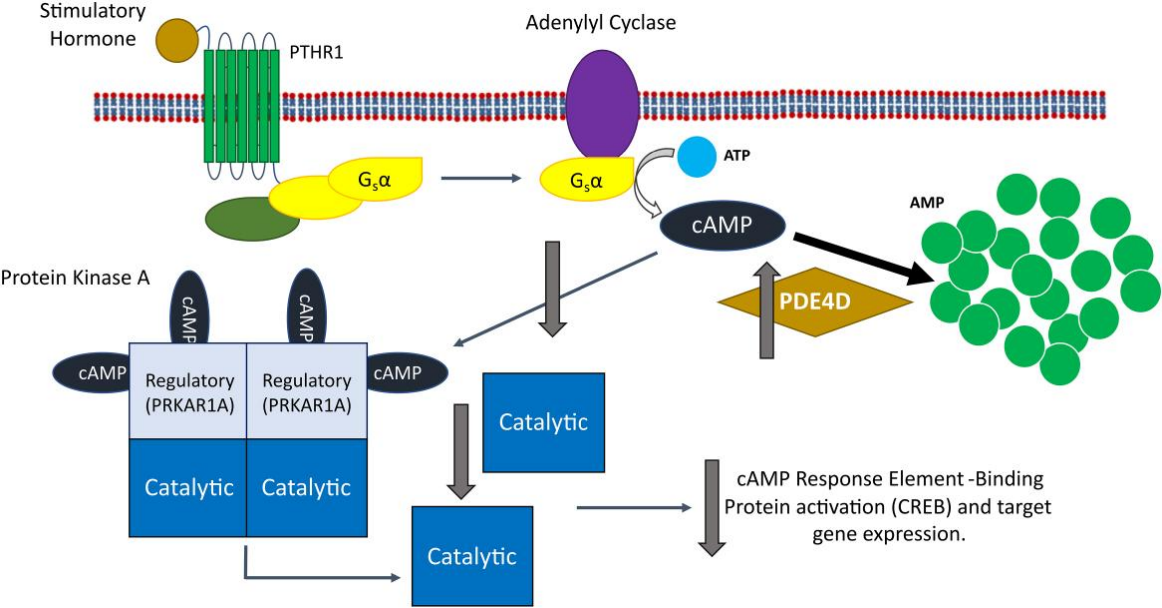
**The impact of the over-activation hypothesis for iPPSD5 on the cAMP/PKA signalling pathway.** The over-activation hypothesis suggests that an increase in PDE4D activity leads to the increased hydrolysis of cAMP to AMP. This removal of cAMP from the signalling pathway leads to reduced levels of free cAMP available to bind and activate PKA, resulting in reduced CREB activation by PKA and therefore a reduction in signalling through the pathway, causing iPPSD5. AMP, adenosine monophosphate; ATP, adenosine triphosphate; cAMP, cyclic adenosine monophosphate; CREB, cAMP response element-binding protein; iPPSD5, inactivating parathyroid hormone/parathyroid hormone-related protein signalling disorder 5; G_s_α, alpha subunit of stimulatory G-protein; PDE, phosphodiesterase; PDE4D, cAMP-specific 3′,5′-cyclic phosphodiesterase 4D; PKA, protein kinase A; PRKAR1A, cAMP-dependent protein kinase type I-alpha regulatory subunit; PTHR1, parathyroid hormone receptor 1.

**Figure 5:**
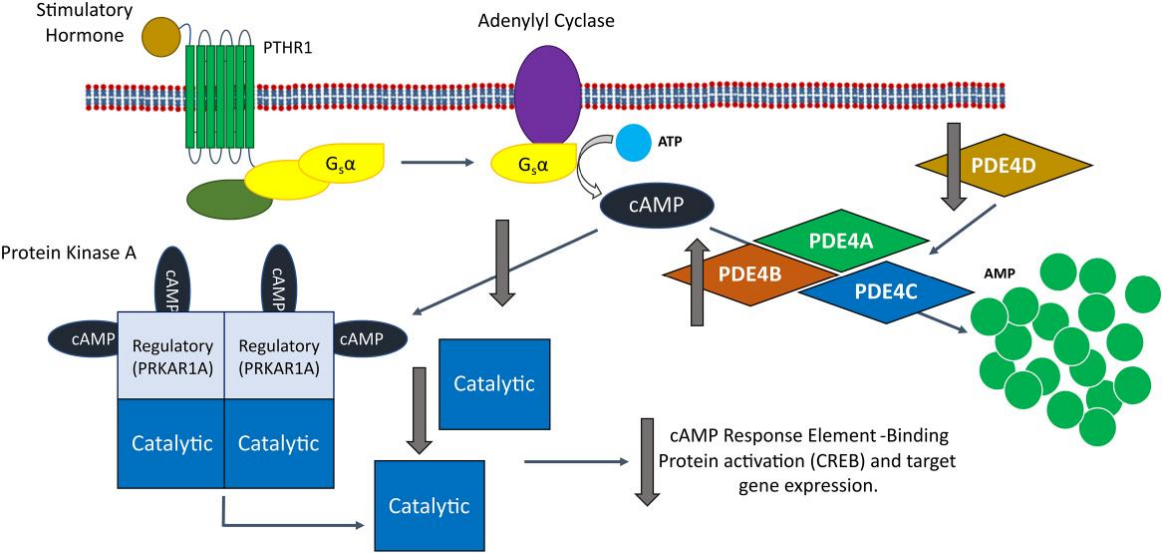
**The impact of the over-compensation hypothesis for iPPSD5 on the cAMP/PKA signalling pathway.** The over-compensation hypothesis suggests that a decrease in PDE4D activity leads to a build-up of cAMP, which triggers the over production of other PDE4 isoforms to compensate for the loss of PDE4D. This leads to the increased removal of cAMP from the signalling pathway, reducing the activation of PKA and reducing CREB activation by PKA. Overall, this leads to a reduction in PKA signalling, leading to iPPSD5. AMP, adenosine monophosphate; ATP, adenosine triphosphate; cAMP, cyclic adenosine monophosphate; CREB, cAMP response element-binding protein; iPPSD5, inactivating parathyroid hormone/parathyroid hormone-related protein signalling disorder 5; G_s_α, alpha subunit of stimulatory G-protein; PDE, phosphodiesterase; PDE4A, cAMP-specific 3′,5′-cyclic phosphodiesterase 4A; PDE4B, cAMP-specific 3′,5′-cyclic phosphodiesterase 4B; PDE4C, cAMP-specific 3′,5′-cyclic phosphodiesterase 4C; PDE4D, cAMP-specific 3′,5′-cyclic phosphodiesterase 4D; PKA, protein kinase A; PRKAR1A, cAMP-dependent protein kinase type I-alpha regulatory subunit; PTHR1, parathyroid hormone receptor 1.

Superficially, there is also an obvious link between mutations in PDE4D and reduced PKA signalling, as an activating (gain of function) mutation in PDE4D would lead to increased hydrolysis of cAMP and therefore reduced PKA activation. However, analysis of the activity of PDE4Ds carrying iPPSD5 causing mutations has been shown to both increase^50^ and decrease activity,^32^ and the only work performed using iPPSD5 patient cells showed no effect on overall PDE or PDE4 activity.^32^ This has led to the presentation of two separate disease mechanisms for iPPSD5.^49^ The first, which we refer to as the ‘over-activation’ hypothesis (Fig. 4), is that mutations in PDE4D result in increased enzymatic activity, leading to a reduction in cAMP levels and consequently reduced PKA signalling.^35,50^ The second, the ‘over-compensation’ hypothesis (Fig. 5), is that mutations in PDE4D lead to reduced activity and therefore an increase in intracellular cAMP. This cAMP build-up is suggested to induce upregulation of other PDE isoforms to compensate for reduced PDE4D activity. In turn, that upregulation leads to an over reduction of cAMP, and the consequent pathological reduction in PKA signalling that would result in iPPSD5.^32^

Where these two hypotheses fundamentally differ is on the effect that disease-causing mutations have on the activity of PDE4D. We will consider the evidence and support that has been suggested for both and suggest an alternative explanation for the role of PDE4D in iPPSD5.

## The over-activation hypothesis

Shortly after the initial identification of PDE mutations in iPPSD5 patients, Lindstrand *et al*.^35^ suggested that because of both the similarity of symptoms to related conditions and the contrasting symptoms in patients with 5q12.1-haploinsufficiency syndrome, it was likely that iPPSD resulted from increased activity of PDE4D mutants. In 2017, Briet *et al*.^50^ published the first experimental evidence showing that mutant PDEs have increased catalytic activity. They showed that when they expressed PDE4D3 carrying iPPSD5 mutations in UCR1, UCR 2 or the catalytic domain (the three regions most commonly affect in iPPSD5) in Chinese hamster ovary cells, the mutants were more susceptible to activation by PKA (including at basal levels), leading to faster and increased cAMP breakdown by the mutants^50^ However, the total hydrolytic capacity of the fully activated wild-type enzyme and of mutants was similar.^50^ This suggests that the over-activation leading to reduced PKA signalling is not due to an overall change in PDE4D activity, but rather to a change in how its enzymatic activity is regulated, in this case a predisposition to be activated by PKA. Although, an important caveat to this is that one of the mutants used by Briet *et al*.^50^ contained a substitution of the serine residue in UCR1 that is phosphorylated by PKA (S54A). Despite this change, the S54A mutant performed similarly to other iPPSD5 mutants, suggesting that other mechanisms of PDE activation may be playing a role.

## The over-compensation hypothesis

The evidence for the ‘over-compensation’ theory (Fig. 5) comes from two sources: the first, Cedervall *et al*.,^10^ who suggested that iPPSD5 mutations would render PDE insensitive to PKA activation, which would normally increase PDE4 activity by 2–6-fold^15,16^; and the second, a study by Kaname *et al*.,^32^ which looked *in silico* at the effect of two iPPSD5 mutations within the catalytic site of PDE4D and used simulations to show that there would be reduced activity in these mutants compared the wild type. Crucially, this does not take account of the other domains (e.g. UCRs) or factors that regulate PDE activity (e.g. phosphorylation by PKA). Kaname *et al*.^32^ also looked at the behaviour of iPPSD5 mutant PDEs (with a range of mutations across the N-terminal domain, UCR1 and catalytic domain) that were overexpressed in the HEK293 (Human Embryonic Kidney 293) cell line. This showed that the lysates from HEK293 cells expressing mutant PDE4D had a lower ability to hydrolyze cAMP than those expressing the wild-type enzyme. It also showed that although there was no difference in the cells at basal levels, but when stimulated with forskolin (to increase intracellular cAMP), the mutant PDEs were less able to attenuate the resulting increase in cAMP than the wild type.^32^ This study also includes the only work published to date using iPPSD5 patient cells, rather than cells overexpressing mutant forms of PDE4D.^32^ Lymphocytes (immortalized using Epstein–Barr virus) from one control donor, one iPPSD4 patient (R368*) and one iPPSD5 patient (L230S) were assessed for their PDE activity and cAMP levels. When lymphocytes from an iPPSD4 patient were treated with forskolin (an activator of adenyl cyclase), the resulting increase in total intracellular cAMP was the same as in control cells. However, because of the mutation of PRKAR1A in these cells, that increase in cAMP did not lead to an increase in the amount phosphorylated (and therefore active) CREB downstream. This is consistent with the hypothesis that iPPSD4 mutations in PRKAR1A prevent the activation of PKA, and therefore reduce CREB activation and downstream signalling. In contrast, when cells from an iPPSD5 patient were treated with forskolin, the resulting increase in intracellular cAMP was significantly lower than in control cells. Given the reduced level of cAMP compared to controls and iPPSD4 cells, it would be expected that there would be less signalling through the cAMP/PKA pathway in iPPSD4 cells. However, the total levels of phosphorylated CREB in the iPPSD5 cells were higher in the iPPSD5 than the iPPSD4 cells and only slightly lower than control cells. This shows that at the whole cell level, there was less disruption in cAMP signalling in the iPPSD5 cells than the iPPSD4 cells. These results differ from the results of the overexpression experiments performed by Kaname *et al*.,^32^ where it was shown that there was no change in total PDE activity, or the activity of the PDE4 family in the iPPSD5 mutants compared to the control. However, a number of other PDE isoforms (including PDE4C, PDE4D5 and PDE4D11) were significantly upregulated at gene expression and protein levels in the patient iPPSD5 line (gene expression in the iPPSD4 line was not determined). This finding was the basis for the over-compensation hypothesis, where decreased activity of the mutant form of PDE4D leads to a build-up of cAMP and triggering an over-compensation from other isoforms. This is not the only study to have linked a reduction in PDE4 activity to an upregulation of the expression of specific isoforms. Susuki-Miyata *et al*.^60^ showed that the inhibition of PDE4 activity in primary human airway epithelial cells with roflumilast leads to an increase in PDE4B mRNA as detected by RT-qPCR, suggesting that exogenous inhibition as well as inactivating mutations could be linked to PDE over-compensation.

## Alternative mechanisms of disease

The work that underlies both the over-activation and over-compensation hypotheses has principally focused on establishing the activity of mutant PDE4Ds using overexpression models in cell lines,^32,50^ rather than cells that carry the mutation themselves and are of a type known to be affected in iPPSD5. The only evidence that has so far been gathered from iPPSD5 patient cells^32^ highlighted the crucial difference between the capacity of PDE4 to hydrolyze cAMP in an overexpression model and the actual effect that a mutation has on a cell when it is expressed under its own regulatory systems. The highly coordinated control that is exerted on cAMP dynamics by PDEs depends on compartmentalization and activation of relatively small pools of the enzyme and this is lost in overexpression models or when looking at a whole cell level, but may be key to the underlying disease mechanism. Work by Tejeda *et al*.,^61^ in an overexpression model, has shown that mutations in PDE10A that cause childhood-onset chorea do not alter the activity levels of the enzyme as Briet *et al*.^50^ have shown with PDE4D in iPPSD5, but instead lead to aberrant localization and reduced protein stability. Similarly, work by Cheguru *et al*.,^62^ has shown how different mutations in the same PDE isoform (PDE6 in this case) can disrupt enzyme activity and localization in different ways, but ultimately cause the same disease. This work showed that when different human mutant PDE6Cs, all known to cause autosomal recessive achromatopsia (partial or total absence of colour vision), were expressed in a transgenic *Xenopus* model, enzymes with mutations in the catalytic domain were trafficked normally but showed reduced enzymatic activity, PDE6s with mutations in the GAF domains (found in c**G**MP-specific phosphodiesterases, **a**denylyl cyclases and **F**hlA^63^) were targeted for proteolytic degradation, and mutations in non-conserved regions caused incorrect localization.

Experimental work by Briet *et al*.^50^ has shown that PDE4D carrying iPPSD5 causing mutations showed increased sensitivity to PKA activation. Interestingly, the increased activation of mutants was not completely mitigated when cells were treated with the protein kinase inhibitor H89,^50^ suggesting that other mechanisms of activation may also be at work. For example, it is possible that iPPSD5 mutations also result in a change in dimerization, which could also contribute to increased activation. The long forms of PDE4 are known to exist as dimers,^14,64,65^ whose formation is regulated by interactions between UCR1 and UCR2; this then leads to the auto-inhibition of the catalytic region via interaction between the C-terminal helical region of one monomers UCR2, which limits access of cAMP to the catalytic site of its partner (trans-capping). As a result of the occlusion of the active site, the long PDE4 dimeric form is less active than the monomeric form,^10^ although activity of the dimeric form can be increased through other forms of regulation e.g. activation by PKA.^15,16^ It is conceivable that the clustering of iPPSD5 mutations in the fully folded protein not only renders mutants more sensitive to PKA activation but also leads to changes in dimerization or auto-inhibition, further contributing to the reduction in PKA signalling that ultimately leads to iPPSD5.

These examples highlight the complexity of PDE activity and indicate that it may not be possible to reduce the mechanism of disease to a question of ‘is PDE4D more or less active?’. To fully understand the mechanism of iPPSD5, it will be important to understand the relative importance of the total potential activity of mutated PDE4Ds (i.e. the capacity of the enzyme to break down cAMP in its most active form) and its level of activation in tissues and cells that are affected in the patients. If the regulation of PDE4D by phosphorylation, dimerization or compartmentalization is dramatically changed in iPPSD5 patients, then a 50% reduction^32^ or 100% increase^50^ in activity of mutated PDE4D compared to wild type may not be as important as it initially appears. It also underlines the need for work to be carried out on patient cells, or cells that have undergone gene editing to express iPPSD5 mutants, so that we do not solely focus on total potential enzyme activity, which may not fully represent the behaviour of PDE4 in cells, but also look at localization, stability, cAMP dynamics using localized genetically encoded cAMP reporters, p-CREB ratios and other non-enzymatic PDE4 functions to get a more complete understanding of the disease mechanism.^60^

## Understanding the cause of intellectual disability in iPPSD5

As well the questions around mechanisms of disease, there are also enduring questions about the differences in disease presentation between iPPSD4 and iPSSD5, and in particular, the origin of intellectual disability in iPSSD5, about which virtually nothing is known. IPPSD5 is not the only condition in which PDEs have been associated with neurological disorders. Phosphodiesterase 4D is associated with neurocognitive functions, such as hippocampal neurogenesis, neurodevelopment and learning.^66^ As well as iPPSD5, mutations in PDE4D lead to several other neurological conditions such as Alzheimer’s disease (PDE4D3, 5, 7 and 9),^67^ depression, fragile X syndrome Huntingdon’s disease, Rett syndrome and schizophrenia draw a clear link between brain function and PDEs.^3,68^ Little is known about the role of PDEs/cAMP during patients’ neurodevelopment, but it is clear from the links to diseases and the positive effects of PDE inhibitors on cognitive function that they play an important role in the brain.^53,69,70^ Although the causes of differences between iPPSD4 and 5 are not well understood, it is likely that tissue distribution and signal intensity are both likely to play a role.

PDEs are known to vary greatly in terms of tissue distribution,^71^ with some isoforms, like PDE4D7 expressed ubiquitously throughout the body, whilst others, like PDE4D6 are expressed only in the brain.^72^ PDE4s are well known to be expressed in the brains of rodents, rhesus macaques and humans,^73-75^ but much of what we know about tissue distribution comes from animals, where the distribution of specific PDEs may differ from humans.^72^ One source of information about the distribution in humans is the Human Protein Atlas (HPA),^76^  https://www.proteinatlas.org/). Data from the HPA shows that both PRKAR1A and PDE4D protein can be detected widely human tissues, but single cell transcriptomic analysis highlights a very high level of *PDE4D* transcripts in excitatory neurons and to a lesser extent in inhibitory neurons and glial cells, whilst very low levels of *PRKAR1A* transcripts were detected in any neural cells.^77^ This gives the clearest indication that we have so far that the neural specificity of iPPSD5 may be in part due to the tissue specificity of *PDE4D*. However, it is not enough to simply classify a tissue as expressing *PRKAR1A*/*PDE4D* or not, as individual cell types within a tissue may have variable PDE expression,^75^ or even respond differently to the same ligand. Seminal work by Motte *et al*.^49^ showed that the effect of a single PTH1R agonist at a specific concentration was cell type dependent. This means that even within a single organ, like the brain, mutations in PDE could be having different effects based on the response of the individual cell types, the isoforms of PDE that they express and their specific response to the combination of agonists they are exposed to.

Motte *et al*.^49^ also showed that different PTH1R agonists could induce different amounts of cAMP production, and crucially, that PDE4s are better able to neutralize stimuli that induce a lower level of cAMP production. This work used HEK-293 cells stably overexpressing PTH1R (HEKpthr). When HEKpthr cells were exposed to a strong stimulus, like PTH, the PDEs within them were shown to have little effect on the increase in intracellular cAMP resulting from receptor activation. In contrast, when a less potent activator, such as isoproterenol, was applied, the PDE4s were capable of hydrolyzing the cAMP that was produced, reducing the spike in intracellular cAMP.^49^ The implication of this is that if the activity of PDE4D is increased, or over-compensated, for example in iPPSD5, this may increase the threshold required to generate a response in the signalling pathway. This would particularly affect responses to weak activators of the pathway. This differential response to stimuli of different intensity may contribute to the absence of hormone resistance in iPPSD5, as PTH has been shown to induce a large increase in cAMP synthesis, this may mitigate the effect of mutations in PDE4D.^49^ This would allow for a normal hormone response in iPPSD4 patients in comparison with iPPSD5 patients where mutant PRKAR1A does not show such input sensitivity. This is also interesting in the case of brachydactyly and short stature in acrodysostosis. Both symptoms are likely to result from aberrant endochondral ossification, resulting from disruption in the PTH/PTHrP signalling cascade in the growth plates of the fingers and long bones of the limbs, respectively.^11,78^ However, the bones of the hand are affected in both iPPSD4 and 5, whilst the long bones are more affected in iPPSD4. It has been shown that although they are similar tissues, there are subtly different forms of paracrine regulation in the growth plates of the fingers and long bones of the limbs.^79^ This suggests that although both symptoms are caused by mis-regulation of the growth plate, defined differences in that regulation, e.g. the ability to respond to stimuli of different intensities, may result in growth being affected or not.

## Conclusions

The role of PDE4D in iPPSD5 is undisputable, yet fundamental questions remain about the mechanism and development of this disease. These include the effect of iPPSD5 mutations on the activity of PDE4D, the knock-on effect of mutations within affected cell types and the cause of tissue specific effects such as learning difficulties in iPPSD5. Acrodysostosis is often diagnosed early in life, but symptoms continue to develop as patients grow. This provides a potential window in which the administration of PDE/PKA modifying drugs could improve outcomes for patients and families. PDE inhibitors have been approved to treat several conditions and clinical trials are undergoing for a PDE4D selective compounds.^80-82^ However, our current understanding of the mechanisms behind iPPSD5 is not advanced enough to develop this kind of treatment. In order to improve our understanding, it will be important to develop cell, tissue and organism level models of acrodysostosis as this will allow for the understanding of how iPPSD5 manifests in the affected tissues as well as the changes mutations cause in PDE4D and PRKAR1A activity.

## Data Availability

Data sharing is not applicable to this article as no new data was created or analysed in this study.

## References

[1] Blair CM, Baillie GS. Reshaping cAMP nanodomains through targeted disruption of compartmentalised phosphodiesterase signalosomes. Biochem Soc Trans. 2019;47(5):1405–1414. 31506329 10.1042/BST20190252

[2] Fertig BA, Baillie GS. PDE4-mediated cAMP signalling. J Cardiovasc Dev Dis. 2018;5(1):8. 29385021 10.3390/jcdd5010008 PMC5872356

[3] Delhaye S, Bardoni B. Role of phosphodiesterases in the pathophysiology of neurodevelopmental disorders. Mol Psychiatry. 2021;26(9):4570–4582. 33414502 10.1038/s41380-020-00997-9 PMC8589663

[4] Conti M, Beavo J. Biochemistry and physiology of cyclic nucleotide phosphodiesterases: Essential components in cyclic nucleotide signaling. Annu Rev Biochem. 2007;76:481–511. 17376027 10.1146/annurev.biochem.76.060305.150444

[5] Houslay MD . PDE4 cAMP-specific phosphodiesterases. Prog Nucleic Acid Res Mol Biol. 2001;69:249–315. 11550796 10.1016/s0079-6603(01)69049-4

[6] Wills L, Ehsan M, Whiteley EL, Baillie GS. Location, location, location: PDE4D5 function is directed by its unique N-terminal region. Cell Signal. 2016;28(7):701–705. 26808969 10.1016/j.cellsig.2016.01.008

[7] Hoffmann R, Baillie GS, MacKenzie SJ, Yarwood SJ, Houslay MD. The MAP kinase ERK2 inhibits the cyclic AMP-specific phosphodiesterase HSPDE4D3 by phosphorylating it at Ser579. EMBO J. 1999;18(4):893–903. 10022832 10.1093/emboj/18.4.893 PMC1171182

[8] Bolger GB, Baillie GS, Li X, et al Scanning peptide array analyses identify overlapping binding sites for the signalling scaffold proteins, beta-arrestin and RACK1, in cAMP-specific phosphodiesterase PDE4D5. Biochem J. 2006;398(1):23–36. 16689683 10.1042/BJ20060423 PMC1525009

[9] Bolger G, Michaeli T, Martins T, et al A family of human phosphodiesterases homologous to the dunce learning and memory gene product of *Drosophila melanogaster* are potential targets for antidepressant drugs. Mol Cell Biol. 1993;13(10):6558–6571. 8413254 10.1128/mcb.13.10.6558-6571.1993 PMC364715

[10] Cedervall P, Aulabaugh A, Geoghegan KF, McLellan TJ, Pandit J. Engineered stabilization and structural analysis of the autoinhibited conformation of PDE4. Proc Natl Acad Sci U S A. 2015;112(12):E1414–E1422. 25775568 10.1073/pnas.1419906112 PMC4378417

[11] Michot C, Le Goff C, Blair E, et al Expanding the phenotypic spectrum of variants in PDE4D/PRKAR1A: From acrodysostosis to acroscyphodysplasia. Eur J Hum Genet. 2018;26(11):1611–1622. 30006632 10.1038/s41431-018-0135-1 PMC6189044

[12] Michot C, Le Goff C, Goldenberg A, et al Exome sequencing identifies PDE4D mutations as another cause of acrodysostosis. Am J Hum Genet. 2012;90(4):740–745. 22464250 10.1016/j.ajhg.2012.03.003 PMC3322219

[13] Lee H, Graham JM Jr, Rimoin DL, et al Exome sequencing identifies PDE4D mutations in acrodysostosis. Am J Hum Genet. 2012;90(4):746–751. 22464252 10.1016/j.ajhg.2012.03.004 PMC3322224

[14] Bolger GB, Dunlop AJ, Meng D, et al Dimerization of cAMP phosphodiesterase-4 (PDE4) in living cells requires interfaces located in both the UCR1 and catalytic unit domains. Cell Signal. 2015;27(4):756–769. 25546709 10.1016/j.cellsig.2014.12.009 PMC4371794

[15] Sette C, Conti M. Phosphorylation and activation of a cAMP-specific phosphodiesterase by the cAMP-dependent protein kinase. Involvement of serine 54 in the enzyme activation. J Biol Chem. 1996;271(28):16526–16534. 8663227 10.1074/jbc.271.28.16526

[16] MacKenzie SJ, Baillie GS, McPhee I, et al Long PDE4 cAMP specific phosphodiesterases are activated by protein kinase A-mediated phosphorylation of a single serine residue in Upstream Conserved Region 1 (UCR1). Br J Pharmacol. 2002;136(3):421–433. 12023945 10.1038/sj.bjp.0704743 PMC1573369

[17] Houslay MD, Adams DR. Putting the lid on phosphodiesterase 4. Nat Biotechnol. 2010;28(1):38–40. 20062038 10.1038/nbt0110-38

[18] Grange M, Sette C, Cuomo M, et al The cAMP-specific phosphodiesterase PDE4D3 is regulated by phosphatidic acid binding. Consequences for cAMP signaling pathway and characterization of a phosphatidic acid binding site. J Biol Chem. 2000;275(43):33379–33387. 10938092 10.1074/jbc.M006329200

[19] Bos JL . Epac proteins: Multi-purpose cAMP targets. Trends Biochem Sci. 2006;31(12):680–686. 17084085 10.1016/j.tibs.2006.10.002

[20] Brand T, Schindler R. New kids on the block: The Popeye domain containing (POPDC) protein family acting as a novel class of cAMP effector proteins in striated muscle. Cell Signal. 2017;40:156–165. 28939104 10.1016/j.cellsig.2017.09.015 PMC6562197

[21] Baillie GS, Tejeda GS, Kelly MP. Therapeutic targeting of 3′,5′-cyclic nucleotide phosphodiesterases: Inhibition and beyond. Nat Rev Drug Discov. 2019;18(10):770–796.

[22] Hayes JS, Brunton LL, Mayer SE. Selective activation of particulate cAMP-dependent protein kinase by isoproterenol and prostaglandin E1. J Biol Chem. 1980;255(11):5113–5119. 6154700

[23] Houslay KF, Christian F, MacLeod R, Adams DR, Houslay MD, Baillie GS. Identification of a multifunctional docking site on the catalytic unit of phosphodiesterase-4 (PDE4) that is utilised by multiple interaction partners. Biochem J. 2017;474(4):597–609. 27993970 10.1042/BCJ20160849 PMC5290487

[24] Carnegie GK, Means CK, Scott JD. A-kinase anchoring proteins: From protein complexes to physiology and disease. IUBMB Life. 2009;61(4):394–406. 19319965 10.1002/iub.168 PMC2682206

[25] Mongillo M, McSorley T, Evellin S, et al Fluorescence resonance energy transfer-based analysis of cAMP dynamics in live neonatal rat cardiac myocytes reveals distinct functions of compartmentalized phosphodiesterases. Circ Res. 2004;95(1):67–75. 15178638 10.1161/01.RES.0000134629.84732.11

[26] Zaccolo M, Pozzan T. Discrete microdomains with high concentration of cAMP in stimulated rat neonatal cardiac myocytes. Science. 2002;295(5560):1711–1715. 11872839 10.1126/science.1069982

[27] Thiele S, Mantovani G, Barlier A, et al From pseudohypoparathyroidism to inactivating PTH/PTHrP signalling disorder (iPPSD), a novel classification proposed by the EuroPHP network. Eur J Endocrinol. 2016;175(6):P1–P17. 27401862 10.1530/EJE-16-0107

[28] Maroteaux P, Lamy M. Diagnosis of chondrodystrophic dwarfism in the newborn. Arch Fr Pediatr. 1968;25(3):241–262. 4970273

[29] Robinow M, Pfeiffer RA, Gorlin RJ, et al Acrodysostosis. A syndrome of peripheral dysostosis, nasal hypoplasia, and mental retardation. Am J Dis Child. 1971;121(3):195–203. 5551869

[30] Ozgur-Gunes Y, Le Stunff C, Chedik M, et al Correction of a knock-in mouse model of acrodysostosis with gene therapy using a rAAV9-CAG-human PRKAR1A vector. Gene Ther. 2022;29(7–8):441–448. 34599290 10.1038/s41434-021-00286-2

[31] Elli FM, Bordogna P, de Sanctis L, et al Screening of PRKAR1A and PDE4D in a large Italian series of patients clinically diagnosed with Albright hereditary osteodystrophy and/or pseudohypoparathyroidism. J Bone Miner Res. 2016;31(6):1215–1224. 26763073 10.1002/jbmr.2785

[32] Kaname T, Ki CS, Niikawa N, et al Heterozygous mutations in cyclic AMP phosphodiesterase-4D (PDE4D) and protein kinase A (PKA) provide new insights into the molecular pathology of acrodysostosis. Cell Signal. 2014;26(11):2446–2459. 25064455 10.1016/j.cellsig.2014.07.025

[33] Linglart A, Silve C, Rothenbuhler A. Multiple hormonal resistances: Diagnosis, evaluation and therapy. Ann Endocrinol (Paris). 2015;76(2):98–100. 25913526 10.1016/j.ando.2015.03.029

[34] Lynch DC, Dyment DA, Huang L, et al Identification of novel mutations confirms PDE4D as a major gene causing acrodysostosis. Hum Mutat. 2013;34(1):97–102. 23033274 10.1002/humu.22222

[35] Lindstrand A, Grigelioniene G, Nilsson D, et al Different mutations in PDE4D associated with developmental disorders with mirror phenotypes. J Med Genet. 2014;51(1):45–54. 24203977 10.1136/jmedgenet-2013-101937

[36] Ertl DA, Mantovani G, de Nanclares GP, et al Growth patterns and outcomes of growth hormone therapy in patients with acrodysostosis. J Endocrinol Invest. 2023;46(8):1673–1684. 36749450 10.1007/s40618-023-02026-2

[37] Hoppmann J, Gesing J, Silve C, et al Phenotypic variability in a family with acrodysostosis type 2 caused by a novel PDE4D mutation affecting the serine target of protein kinase-a phosphorylation. J Clin Res Pediatr Endocrinol. 2017;9(4):360–365. 28515031 10.4274/jcrpe.4488 PMC5785644

[38] Linglart A, Menguy C, Couvineau A, et al Recurrent PRKAR1A mutation in acrodysostosis with hormone resistance. N Engl J Med. 2011;364(23):2218–2226. 21651393 10.1056/NEJMoa1012717

[39] Martin TJ . PTH1R actions on bone using the cAMP/protein kinase a pathway. Front Endocrinol (Lausanne). 2021;12:833221. 35126319 10.3389/fendo.2021.833221 PMC8807523

[40] Mantovani G, Elli FM. Multiple hormone resistance and alterations of G-protein-coupled receptors signaling. Best Pract Res Clin Endocrinol Metab. 2018;32(2):141–154. 29678282 10.1016/j.beem.2018.01.002

[41] Rock R, Mayrhofer JE, Bachmann V, Stefan E. Impact of kinase activating and inactivating patient mutations on binary PKA interactions. Front Pharmacol. 2015;6:170. 26347651 10.3389/fphar.2015.00170 PMC4539479

[42] Bruystens JG, Wu J, Fortezzo A, et al Structure of a PKA RIalpha recurrent acrodysostosis mutant explains defective cAMP-dependent activation. J Mol Biol. 2016;428(24 Pt B):4890–4904. 27825928 10.1016/j.jmb.2016.10.033 PMC5149412

[43] Gonzalez GA, Montminy MR. Cyclic AMP stimulates somatostatin gene transcription by phosphorylation of CREB at serine 133. Cell. 1989;59(4):675–680. 2573431 10.1016/0092-8674(89)90013-5

[44] Nichols M, Weih F, Schmid W, et al Phosphorylation of CREB affects its binding to high and low affinity sites: Implications for cAMP induced gene transcription. EMBO J. 1992;11(9):3337–3346. 1354612 10.1002/j.1460-2075.1992.tb05412.x PMC556868

[45] Mantovani G, Elli FM. Inactivating PTH/PTHrP signaling disorders. Front Horm Res. 2019;51:147–159. 30641531 10.1159/000491045

[46] Silve C . Acrodysostosis: A new form of pseudohypoparathyroidism? Ann Endocrinol (Paris). 2015;76(2):110–112. 25890446 10.1016/j.ando.2015.03.004

[47] Nagasaki K, Iida T, Sato H, et al PRKAR1A mutation affecting cAMP-mediated G protein-coupled receptor signaling in a patient with acrodysostosis and hormone resistance. J Clin Endocrinol Metab. 2012;97(9):E1808–E1813. 22723333 10.1210/jc.2012-1369

[48] Rhayem Y, Le Stunff C, Abdel Khalek W, et al Functional characterization of PRKAR1A mutations reveals a unique molecular mechanism causing acrodysostosis but multiple mechanisms causing Carney complex. J Biol Chem. 2015;290(46):27816–27828. 26405036 10.1074/jbc.M115.656553 PMC4646027

[49] Motte E, Le Stunff C, Briet C, Dumaz N, Silve C. Modulation of signaling through GPCR-cAMP-PKA pathways by PDE4 depends on stimulus intensity: Possible implications for the pathogenesis of acrodysostosis without hormone resistance. Mol Cell Endocrinol. 2017;442:1–11. 27908835 10.1016/j.mce.2016.11.026

[50] Briet C, Pereda A, Le Stunff C, et al Mutations causing acrodysostosis-2 facilitate activation of phosphodiesterase 4D3. Hum Mol Genet. 2017;26(20):3883–3894. 29016851 10.1093/hmg/ddx271

[51] Venkatakrishnan V, Ghode A, Tulsian NK, Anand GS. Impaired cAMP processivity by phosphodiesterase-protein kinase A complexes in acrodysostosis. Front Mol Biosci. 2023;10:1202268. 37808519 10.3389/fmolb.2023.1202268 PMC10552185

[52] Maass PG, Aydin A, Luft FC, et al PDE3A mutations cause autosomal dominant hypertension with brachydactyly. Nat Genet. 2015;47(6):647–653. 25961942 10.1038/ng.3302

[53] Blokland A, Van Duinen MA, Sambeth A, et al Acute treatment with the PDE4 inhibitor roflumilast improves verbal word memory in healthy old individuals: A double-blind placebo-controlled study. Neurobiol Aging. 2019;77:37–43. 30776650 10.1016/j.neurobiolaging.2019.01.014

[54] Knott EP, Assi M, Rao SN, Ghosh M, Pearse DD. Phosphodiesterase inhibitors as a therapeutic approach to neuroprotection and repair. Int J Mol Sci. 2017;18(4):696. 28338622 10.3390/ijms18040696 PMC5412282

[55] Prickaerts J, Heckman PRA, Blokland A. Investigational phosphodiesterase inhibitors in phase I and phase II clinical trials for Alzheimer’s disease. Expert Opin Investig Drugs. 2017;26(9):1033–1048. 10.1080/13543784.2017.1364360 28772081

[56] Van Duinen MA, Sambeth A, Heckman PRA, et al Acute administration of roflumilast enhances immediate recall of verbal word memory in healthy young adults. Neuropharmacology. 2018;131:31–38. 29241652 10.1016/j.neuropharm.2017.12.019

[57] Goltzman D . Physiology of parathyroid hormone. Endocrinol Metab Clin North Am. 2018;47(4):743–758. 30390810 10.1016/j.ecl.2018.07.003

[58] Vortkamp A, Lee K, Lanske B, Segre GV, Kronenberg HM, Tabin CJ. Regulation of rate of cartilage differentiation by Indian hedgehog and PTH-related protein. Science. 1996;273(5275):613–622. 8662546 10.1126/science.273.5275.613

[59] Kronenberg HM . Developmental regulation of the growth plate. Nature. 2003;423(6937):332–336. 12748651 10.1038/nature01657

[60] Susuki-Miyata S, Miyata M, Lee BC, et al Cross-talk between PKA-Cbeta and p65 mediates synergistic induction of PDE4B by roflumilast and NTHi. Proc Natl Acad Sci U S A. 2015;112(14):E1800–E1809. 25831493 10.1073/pnas.1418716112 PMC4394257

[61] Tejeda GS, Whiteley EL, Deeb TZ, et al Chorea-related mutations in PDE10A result in aberrant compartmentalization and functionality of the enzyme. Proc Natl Acad Sci U S A. 2020;117(1):677–688. 31871190 10.1073/pnas.1916398117 PMC6955301

[62] Cheguru P, Majumder A, Artemyev NO. Distinct patterns of compartmentalization and proteolytic stability of PDE6C mutants linked to achromatopsia. Mol Cell Neurosci. 2015;64:1–8. 25461672 10.1016/j.mcn.2014.10.007 PMC4323879

[63] Ho YS, Burden LM, Hurley JH. Structure of the GAF domain, a ubiquitous signaling motif and a new class of cyclic GMP receptor. EMBO J. 2000;19(20):5288–5299. 11032796 10.1093/emboj/19.20.5288 PMC314001

[64] Richter W, Conti M. Dimerization of the type 4 cAMP-specific phosphodiesterases is mediated by the upstream conserved regions (UCRs). J Biol Chem. 2002;277(43):40212–40221. 12177055 10.1074/jbc.M203585200

[65] Xie M, Blackman B, Scheitrum C, et al The upstream conserved regions (UCRs) mediate homo- and hetero-oligomerization of type 4 cyclic nucleotide phosphodiesterases (PDE4s). Biochem J. 2014;459(3):539–550. 24555506 10.1042/BJ20131681 PMC4315173

[66] Li YF, Cheng YF, Huang Y, et al Phosphodiesterase-4D knock-out and RNA interference-mediated knock-down enhance memory and increase hippocampal neurogenesis via increased cAMP signaling. J Neurosci. 2011;31(1):172–v183. 21209202 10.1523/JNEUROSCI.5236-10.2011 PMC3079568

[67] Paes D, Schepers M, Willems E, et al Ablation of specific long PDE4D isoforms increases neurite elongation and conveys protection against amyloid-beta pathology. Cell Mol Life Sci. 2023;80(7):178. 37306762 10.1007/s00018-023-04804-w PMC10261250

[68] Choi CH, Schoenfeld BP, Weisz ED, et al PDE-4 inhibition rescues aberrant synaptic plasticity in Drosophila and mouse models of fragile X syndrome. J Neurosci. 2015;35(1):396–408. 25568131 10.1523/JNEUROSCI.1356-12.2015 PMC4287155

[69] Wu Y, Li Z, Huang YY, Wu D, Luo HB. Novel phosphodiesterase inhibitors for cognitive improvement in Alzheimer’s disease. J Med Chem. 2018;61(13):5467–5483. 29363967 10.1021/acs.jmedchem.7b01370

[70] Gilleen J, Farah Y, Davison C, et al An experimental medicine study of the phosphodiesterase-4 inhibitor, roflumilast, on working memory-related brain activity and episodic memory in schizophrenia patients. Psychopharmacology (Berl). 2021;238(5):1279–1289. 30536081 10.1007/s00213-018-5134-y PMC8062361

[71] Richter W, Jin SL, Conti M. Splice variants of the cyclic nucleotide phosphodiesterase PDE4D are differentially expressed and regulated in rat tissue. Biochem J. 2005;388(Pt 3):803–811. 15717866 10.1042/BJ20050030 PMC1183459

[72] Wang D, Deng C, Bugaj-Gaweda B, et al Cloning and characterization of novel PDE4D isoforms PDE4D6 and PDE4D7. Cell Signal. 2003;15(9):883–891. 12834813 10.1016/s0898-6568(03)00042-1

[73] Engels P, Abdel'Al S, Hulley P, Lubbert H. Brain distribution of four rat homologues of the Drosophila dunce cAMP phosphodiesterase. J Neurosci Res. 1995;41(2):169–178. 7650752 10.1002/jnr.490410204

[74] Bolger GB, Rodgers L, Riggs M. Differential CNS expression of alternative mRNA isoforms of the mammalian genes encoding cAMP-specific phosphodiesterases. Gene. 1994;149(2):237–244. 7958996 10.1016/0378-1119(94)90155-4

[75] Datta D, Enwright JF, Arion D, et al Mapping phosphodiesterase 4D (PDE4D) in macaque dorsolateral prefrontal cortex: Postsynaptic compartmentalization in layer III pyramidal cell circuits. Front Neuroanat. 2020;14:578483. 33328902 10.3389/fnana.2020.578483 PMC7714912

[76] Uhlen M, Fagerberg L, Hallstrom BM, et al Proteomics. Tissue-based map of the human proteome. Science. 2015;347(6220):1260419. 25613900 10.1126/science.1260419

[77] Sjöstedt E, Zhong W, Fagerberg L, et al An atlas of the protein-coding genes in the human, pig, and mouse brain. Science. 2020;367(6482):1090. 10.1126/science.aay5947 32139519

[78] Silve C, Clauser E, Linglart A. Acrodysostosis. Horm Metab Res. 2012;44(10):749–758. 22815067 10.1055/s-0032-1316330

[79] Zhou E, Lui J. Physiological regulation of bone length and skeletal proportion in mammals. Exp Physiol. 2021;106(2):389–395. 33369789 10.1113/EP089086 PMC7855611

[80] Blauvelt A, Langley RG, Gordon KB, et al Next generation PDE4 inhibitors that selectively target PDE4B/D subtypes: A narrative review. Dermatol Ther (Heidelb). 2023;13(12):3031–3042. 37924462 10.1007/s13555-023-01054-3 PMC10689637

[81] Berry-Kravis EM, Harnett MD, Reines SA, et al Inhibition of phosphodiesterase-4D in adults with fragile X syndrome: A randomized, placebo-controlled, phase 2 clinical trial. Nat Med. 2021;27(5):862–870. 33927413 10.1038/s41591-021-01321-w

[82] Bondarev AD, Attwood MM, Jonsson J, et al Recent developments of phosphodiesterase inhibitors: Clinical trials, emerging indications and novel molecules. Front Pharmacol. 2022;13:1057083. 36506513 10.3389/fphar.2022.1057083 PMC9731127

[83] Zhan Y, Chen W, Feng Z, et al A novel de novo PDE4D gene mutation identified in a Chinese patient with acrodysostosis. Genesis. 2019;57(11–12):e23336. 31520578 10.1002/dvg.23336

[84] Petraityte G, Siauryte K, Mikstiene V, et al A novel variant in the PDE4D gene is the cause of Acrodysostosis type 2 in a Lithuanian patient: A case report. BMC Endocr Disord. 2021;21(1):71. 33858404 10.1186/s12902-021-00741-6 PMC8051037

